# Classification and microsurgical treatment of foramen magnum meningioma

**DOI:** 10.1186/s41016-022-00315-y

**Published:** 2023-01-24

**Authors:** Pengfei Wu, Yanlei Guan, Minghao Wang, Luyang Zhang, Dan Zhao, Xiao Cui, Jiyuan Liu, Bo Qiu, Jun Tao, Yunjie Wang, Shaowu Ou

**Affiliations:** Department of Neurosurgery, the First Affiliated Hospital, China Medical University, 155 Nanjing Street, Heping District, Shenyang, 110001 Liaoning China

**Keywords:** Classification, Anatomical triangles, Foramen magnum meningioma, Microsurgical treatment, Far lateral approach

## Abstract

**Background:**

To investigate the classification and microsurgical treatment of foramen magnum meningioma (FMM).

**Methods:**

We retrospectively analyzed 76 patients with FMM and classified them into two classifications, classification ABS according to the relationship between the FMM and the brainstem and classification SIM according to the relationship between the FMM and the vertebral artery (VA). All patients underwent either the far lateral approach (54 cases) or the suboccipital midline approach (22 cases).

**Results:**

Of the 76 cases, 47 cases were located ahead of the brainstem (A), 16 cases at the back of the brainstem (B), and 13 cases were located laterally to the brainstem (S). There were 15 cases located superior to the VA (S), 49 cases were inferior (I), and 12 cases were mixed type (M). Among 76 cases, 71 cases were resected with Simpson grade 2 (93.42%), 3 with Simpson grade 3 (3.95%), and 2 with Simpson grade 4 (2.63%). We summarized four anatomical triangles: triangles SOT, VOT, JVV, and TVV. The mean postoperative Karnofsky performance score was improved in all patients (*p* < 0.05). However, several complications occurred, including hoarseness and CSF leak.

**Conclusion:**

ABS and SIM classifications are objective indices for choosing the surgical approach and predicting the difficulty of FMMs, and it is of great importance to master the content, position relationship with the tumor, and variable anatomical structures in the four “triangles” for the success of the operation.

## Background

The foramen magnum region is an important passage from the brain to the spinal cord and is the continuation of the central nervous system. It is also the only way through which many blood vessels and peripheral nerves pass and is the main site of some nervous system tumors [1–8].

The anatomical structure in this region is very complex, causing many difficulties in surgical operation. Many anatomical and clinical studies on the operation of foramen magnum lesions have been reported [9–11]. Far lateral approach (FLA) is the extension and expansion of the standard retrosigmoid approach and is mainly used to expose the ventral side of the brainstem and cervical spinal cord maximally and the vast space from the clivus to C2 and to reveal the relationship between tumors and the medulla oblongata and cervical spinal cord from the coronal. Important structures, such as the medulla oblongata and cervical spinal cord, do not need to be retracted to expose and remove FMMs effectively [12–19]. However, its clinical application is severely limited by the complicated anatomical relationships of important neurovascular structures, the deep location of the lesions, the high risk of intraoperative injury, and slow postoperative recovery. This study retrospectively analyzed 76 patients with foramen magnum meningioma (FMM) at our hospital. We provide two classifications for FMM and summarize the four anatomical “triangles” for the critical parts to be paid attention to during the operation and hope it will be helpful and referential to the operation of this region.

## Methods

### Subjects

We retrospectively analyzed the clinical data of 76 patients with FMM treated with microsurgery in our department from January 2010 to September 2019. The patients included in this study were 25 males and 51 females, aged 23 to 72 years old, with an average age of 47.32 ± 18.51 years old. The main clinical manifestations included 28 cases of neck and occipital pain, 22 cases of decreased muscle strength and hypoesthesia of the limb, 11 cases of dysphagia, seven cases of intracranial hypertension, five cases of ataxia, and three cases of dyspnea (Table 1). Karnofsky performance score (KPS) was used to evaluate the functional status of the patients, and the mean preoperative KPS score was 76.3 ± 12.8. All patients were followed up for 3 to 106 months (mean 53.92 ± 21.57 months).

**Table 1:** The clinical characteristics of foramen magnum meningioma (FMM)

	Content		Cases	Percentage (%)
Gale	Male		25	32.89
	Female		51	67.11
The type of position of FMM	Type ABS (around the brain stem)	Ahead of the brainstem (A)	47	61.84
		Back of the brainstem (B)	8	10.53
		Side of the brainstem (S)	21	27.63
	Type SIM (around the vertebral artery)	Superior to the vertebral artery (S)	19	25.00
		Inferior to the vertebral artery (I)	39	51.32
		Mixed of the vertebral artery (M)	18	23.68
Manifestations	Neck and occipital pain		28	36.84
	Decreased limb muscle strength and hypoesthesia		22	28.95
	Dysphagia and drinking water choking cough		11	14.47
	Increased intracranial pressure		7	9.21
	Ataxia		5	6.58
	Dyspnea		3	3.95
Operative approaches	Far lateral approach	Ahead of the brainstem (A)	47	77.63
		Side of the brainstem (S)	12	
	Suboccipital midline approach	Side of the brainstem (S)	9	22.37
		Back of the brainstem (B)	8	
Postoperative complications	Hoarseness, drinking water choking cough, dysphagia		7	9.21
	Hypodermic fluidification		5	6.58
	Pneumonia		4	5.26
	Weak sputum and cough reflex		3	3.95
	Neck and occipital numbness		3	3.95
	Incision infection		3	3.95
	Great occipital neuralgia		2	2.63
	Dyspnea		2	2.63
	Cerebrospinal fluid leakage		2	2.63
	Intracranial infection		2	2.63
	Hydrocephalus		2	2.63
	Contralateral limb strength decreased		1	1.32

All patients underwent computed tomography (CT) (AQI 64 slice spiral CT, Toshiba, JAN), magnetic resonance imaging (MRI) (Mobile Signa Advantage 1.5T or 3.0T, GE Healthcare, USA), three-dimensional computer tomography angiography (3DCTA) (AQI 64 slice spiral CT, Toshiba, JAN), and sometimes digital subtraction angiography (DSA) (Innova 3100IQ, GE Healthcare, USA) evaluations.

The tumors ranged in size from 1.2 × 1.5 × 2.7 cm^3^ to 2.1 × 2.6 × 5.1 cm^3^ (average, 1.7 × 2.0 × 3.3 cm^3^). MRI and contrast MRI showed isointensity in the longitudinal relaxation time-weighted images (T1W) and transversal relaxation time-weighted images (T2W), and the tumors were enhanced, and the dural tail sign could be seen in the MRI contrast sequence. CTA revealed increased blood and tumors, as well as a relationship between them. DSA is usually used to distinguish meningiomas from solid hemangioblastomas at the dorsal medulla oblongata.

### Classifications

To facilitate the choice of surgical approach and predict the difficulty of FMM resection, we provide two classifications for FMM according to the relationship between the FMM and the brainstem and vertebral artery (VA).

**Fig. 1:**
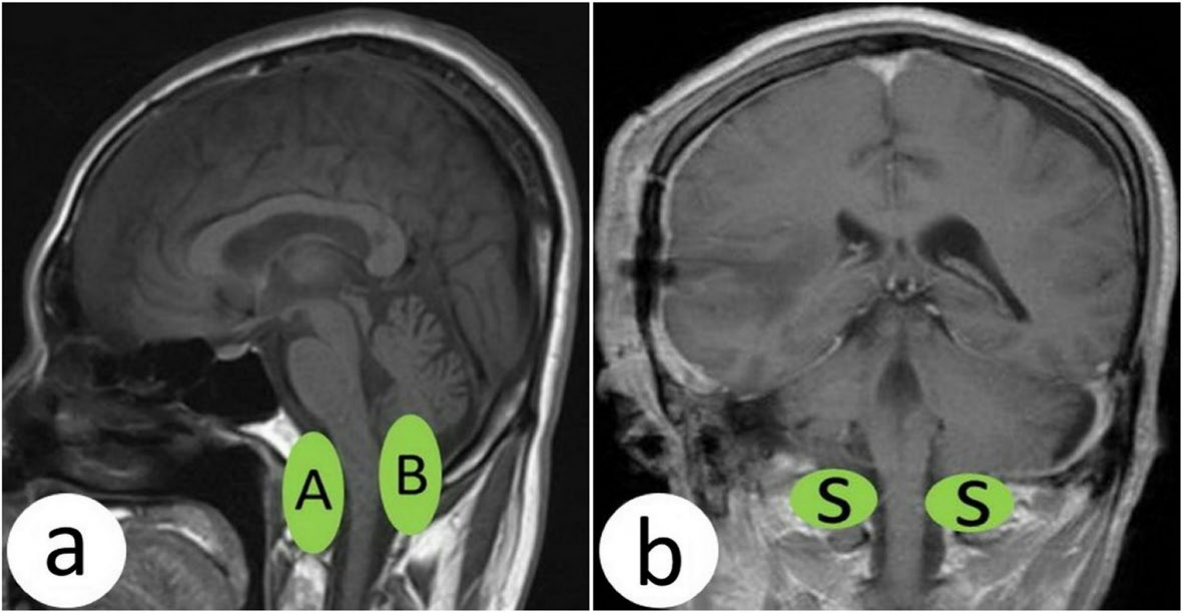
The relationship between FMM and brainstem. **a** A, The FMM locates ahead of the brainstem; B, the FMM locates in the back of the brainstem. **b** S, the FMM locates the side of the brainstem

**Fig. 2:**
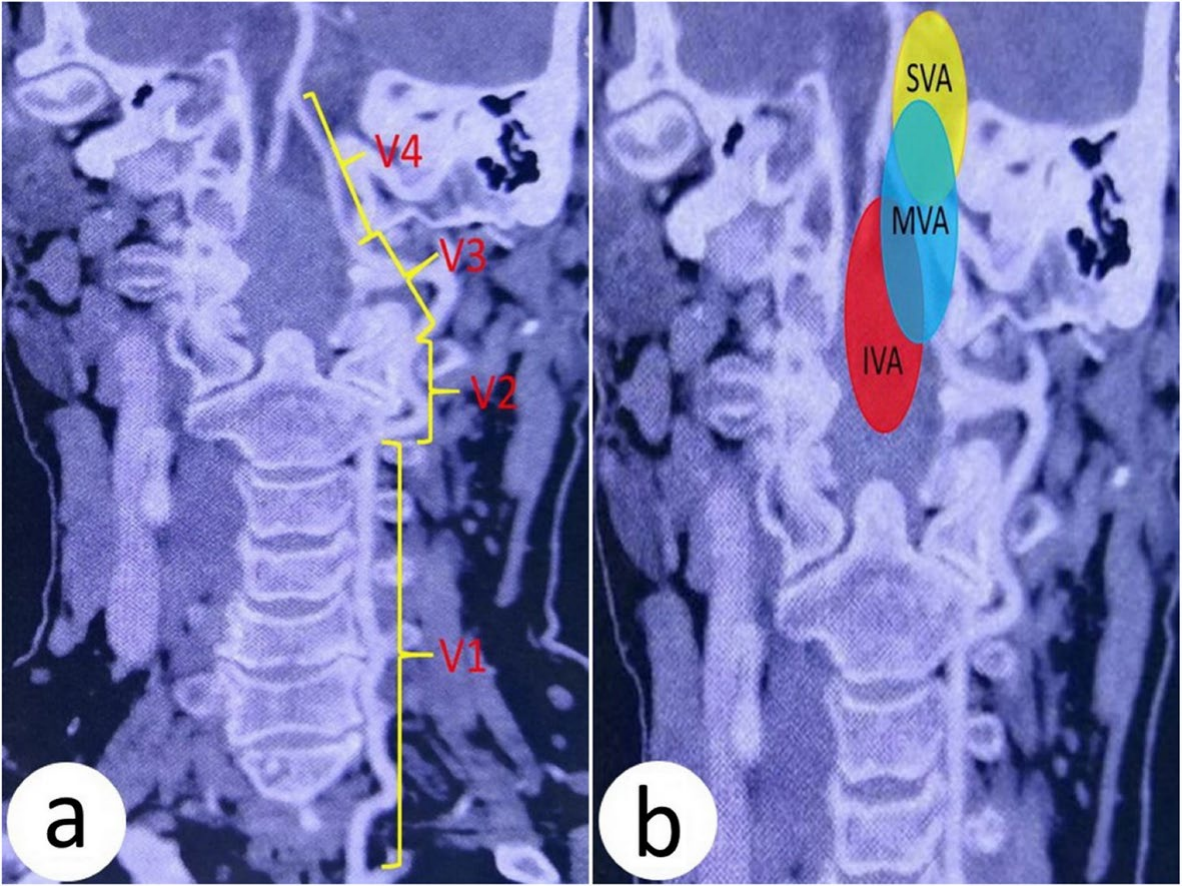
The segment of the vertebral artery and the relationship between FMM and vertebral artery. **a** The segment of the vertebral artery. V1, from the origin of the vertebral artery to the upper margin of the lateral foramen of C2. V2, from the upper margin of the lateral foramen of C2 to the lower margin of the transverse foramen C1. V3, from the lower margin of the transverse foramen of C1 to the vertebral artery craniotomy. V4, intracranial segment of the vertebral artery. **b** The relationship between FMM and V4. SVA, IVA, and MVA, the FMM is located in the superior, inferior, and mixed vertebral artery

1. ABS: Based on the relationship between the FMM and brainstem, we classified it into ahead of the brainstem (A), back of the brainstem (B), and side of the brainstem (S) (Fig. 1).
2. SIM: According to the position relationship between the FMM and the VA, it can be classified as follows: superior to the VA (S), inferior to the VA (I), and mixed (M) (Fig. 2).

**Fig. 3:**
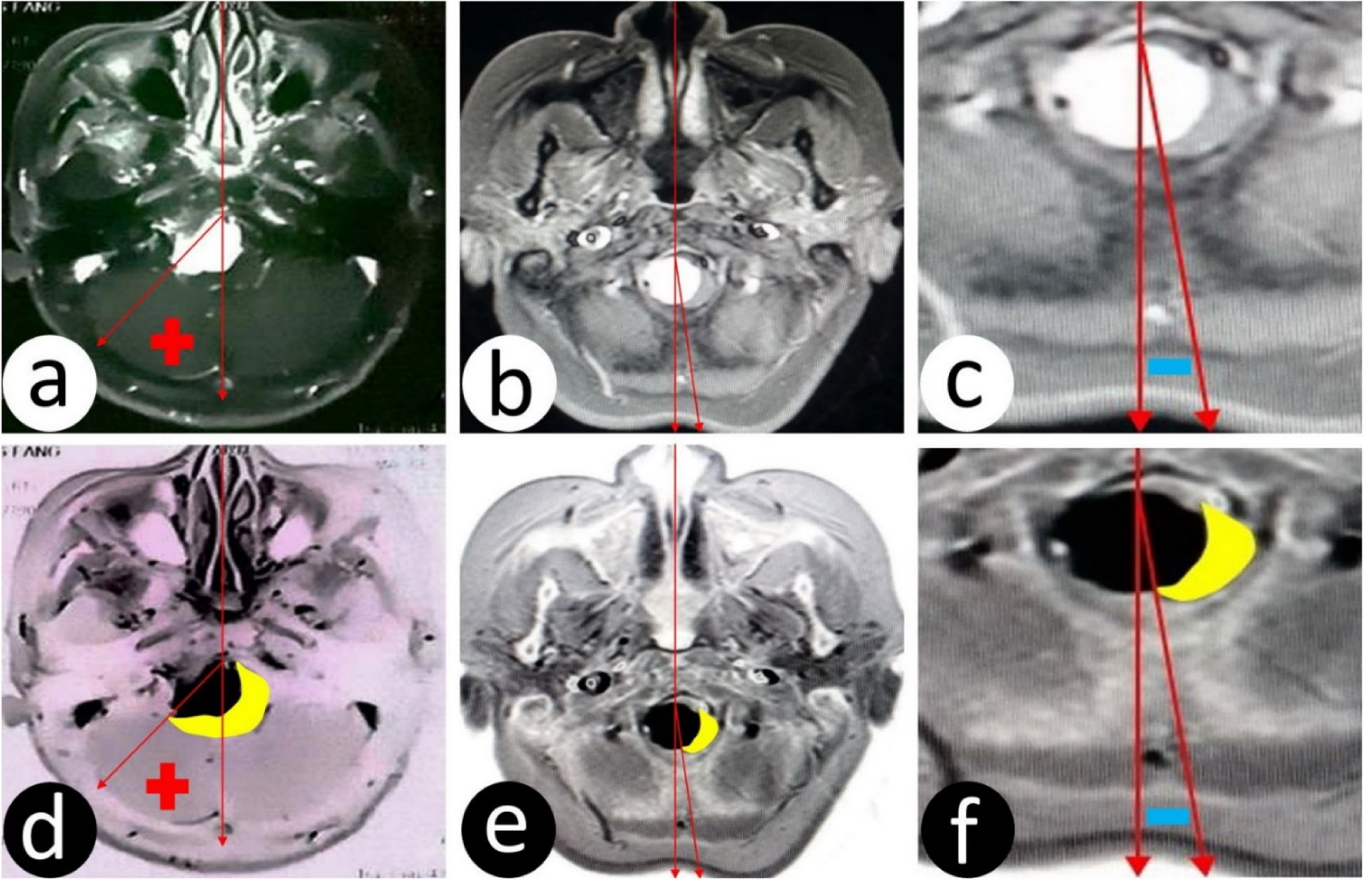
How to define whether a tumor is ahead or on the side of the brainstem? **a** The maximum level axis tumor enhancement MRI is divided into two equal parts by the median sagittal line, and the pressed brainstem with a lateral displacement is across the median sagittal plane to the contralateral; tumor is believed to be ahead of the brainstem, expressed in the red “+.” **b** The maximum level axis tumor enhancement MRI is divided into two equal parts by the median sagittal line, and the pressed brainstem with a lateral displacement is not across the median sagittal plane to the contralateral; the tumor is believed to be the side of the brainstem. In the local amplification of **b**, the tumor is believed to be the side of the brainstem, expressed in the blue “−.” **d**–**f** The film in **a**–**c** and the yellow part is the brainstem

How to define whether a tumor is in front of or lateral to the brainstem? We divide the MRI image of the maximum axial level of the tumor into two equal parts using a median vertical line; if the brainstem is pressed to a lateral displacement and across the median vertical line, the tumor is believed to be in front of the brainstem, expressed in the red “+”; if the pressed brainstem is not across the median vertical line to the contralateral, the tumor is believed to be on lateral to the brainstem, expressed in the blue “−” (Fig. 3a–f).

### Choice of surgical approach and microsurgery

#### Choice of surgical approach

The FLA or suboccipital midline approach was performed according to the location and classification of the tumor. Generally speaking, an FLA could be chosen when an FMM is located ahead of the brainstem (A), a suboccipital midline approach could be chosen when an FMM is located at the back of the brainstem (B), and an FLA or a suboccipital midline approach could be chosen when an FMM is located side of the brainstem (S). However, the ABS and SIM classifications are not independent; an FMM could be ABS and superior, inferior, or mixed location to the VA, and the approach chosen is also different according to different situations (Table 2).

**Table 2:** The classification and choice of surgical approach of the 76 foramen magnum meningiomas

Classification ABS	Cases	Classification SIM	Cases	Surgical approach	Cases
A	47	S	10	Paracondylar far lateral approach^a^	5
				Basic far lateral approach	3
				Supracondylar far lateral approach extension	2
		I	29	Basic far lateral approach	21
				Transcondylar far lateral approach extension	8
		M	8	Basic far lateral approach	4
				Transcondylar far lateral approach extension	2
				Supracondylar far lateral approach extension	2
B	8	S	4	Suboccipital midline approach	8
		I	1		
		M	3		
S	21	S	5	Paracondylar far lateral approach	2
				Basic far lateral approach	2
				Supracondylar far lateral approach extension	1
		I	9	Basic far lateral approach	2
				Suboccipital midline approach	7
		M	7	Basic far lateral approach	5
				Suboccipital midline approach	2

^a^The paracondylar far lateral approach is the same as the basic far lateral approach, which does not remove the posterior atlas tubercle

#### Microsurgery of FLA

**Fig. 4:**
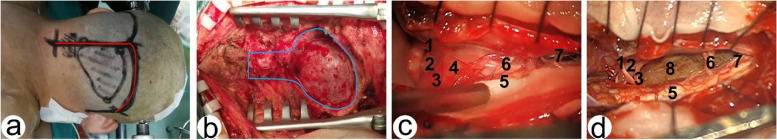
The process of microsurgery of the far lateral approach. **a** The inverted “L”-shaped skin incision (red line). **b** The inverted boot pattern bone window formed (blue line). **c**–**d** Preoperative and postoperative microscopic images. 1, accessory nerve; 2, vertebral artery; 3, C1 nerve; 4, tumor; 5, brainstem; 6, C2 nerve; 7, C3 nerve; 8, tumor bed

Position and skin incision of FLA: The patient was in the lateral prone position, whose head was raised 15° to facilitate the venous return, and the head was forward 10° of flexion and 15° to the side of the neck slightly buckled to make the same side of the temporal bone mastoid and superior nuchal line located at the highest point and to increase the clearance between the foramen magnum and atlas; at the same time, the trouble side shoulder should be pulled in the direction of the feet to increase neck exposure. The head of the patient was fixed on a Mayfield head frame [1], and an inverted “L”-shaped skin incision started at the junction of the transverse sinus and sigmoid sinus, reached the posterior median line along the superior line, and then stopped at the 3rd and 4th cervical spine processes. The skin and subcutaneous tissue were cut open, and the muscular layers were cut and retracted laterally (Fig. 4a).

An inverted boot pattern bone window was formed, including the posterior tubercle of the atlas, and the occipital condyle should be partially resected when necessary. The amount of occipital condyle removal should be determined according to the extent to which the lesion involves the ahead of the brainstem, generally less than one-third (Fig. 4b)

According to the results of preoperative MRI and 3DCTA, the tumor was removed in blocks in the space between the nerves, blood vessels, and brainstem to gradually increase the exposure space. If it is unnecessary, nerves C1, C2, and C3 are not recommended to be cut off; otherwise, obvious postoperative occipital and shoulder discomfort and even diaphragmatic spasm will occur. Because there are more benign tumors in this area, most of them have an obvious arachnoid space with normal brain tissue. The separation of tumors along this space can not only maximally protect normal brain tissue but also completely remove the tumor (Fig. 4c and d).

#### Microsurgery of the suboccipital midline approach

**Fig. 5:**
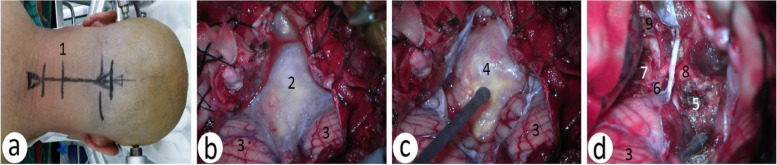
The process of microsurgery of suboccipital midline approach. **a** The incision is performed strictly according to the median. **b** Cisterna magna and cerebellum are exposed. **c** Tumor is exposed. **d** Tumor bed is exposed after the tumor was removed. 1, the incision; 2, the arachnoid membrane of the cisterna magna; 3, cerebellum; 4, tumor; 5, tumor bed; 6, PICA; 7, the roots of the tumor; 8, brainstem; 9, vertebral artery

The patient was placed in the prone position, fixed with a Mayfield head frame, and the medial ligament was cut under the external occipital protuberance to the level of cervical 2, exposing the occipital scales and posterior atlas arch. The upper margin of the bone window exposes the lower margin of the bilateral transverse sinuses, opening of the foramen magnum below, and, if necessary, the posterior arch of the atlas. The conventional “Y”-shaped incision of the dura or “TT”-shaped incision of the occipital sinus can be used to reduce bleeding. The tumor is generally located in the subdural or below the cistern magnum, dorsal to the brainstem. Based on the imaging characteristics, the blood supply at the root of the tumor was blocked under the microscope, and the adhesion and entanglement between the tumor and the brainstem, posterior cranial nerves, cervical nerves, VA, posterior inferior cerebellar artery, and its branches were released and separated, and then, the tumor tissue was completely removed (Fig. 5a–d).

### Statistical analysis

Data were statistically analyzed with SPSS 19.0 statistical software (SPSS, Chicago, IL, USA) and expressed as the mean ± SD and *χ*^2^ inspections.

## Results

### Operation results

After the operation, all patients were intubated back to the neurosurgery intensive care unit (NICU) under spontaneous breathing to prevent decreased blood oxygen saturation due to reduced respiratory function and/or cough reflexes. After head CT review and confirmation of good respiratory function and/or obvious cough reflex, endotrache extubation, the patient was given preventive nasal feeding to prevent aspiration. If there was no obvious cough reflex, tracheotomy should be considered after observation. The mean postoperative KPS score was 89.2 ± 10.7, which improved in all patients (*p* < 0.05).

**Fig. 6:**
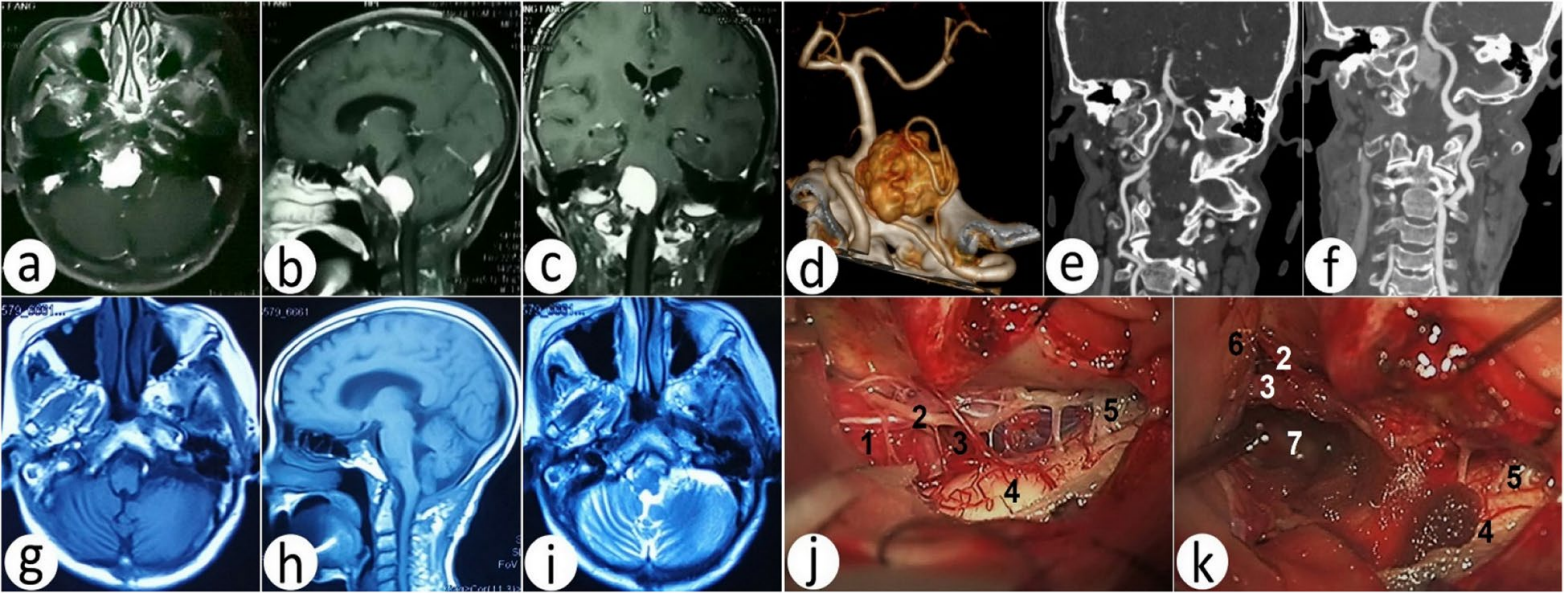
Comparison of preoperative and postoperative images of the FMM, which is located ahead of the brainstem and superior to the vertebral artery. **a**–**c** Preoperative axial, sagittal, and coronal T1-weighted contrast enhanced. **d** Preoperative 3DCTA and tumor image reconstruction. **e**–**f** Preoperative right and left vertebral artery 3DCTA image reconstruction. **g**–**h** Postoperative axial and sagittal T1 weighted. **i** Postoperative axial T2 weighted. **j** Preoperative tumor, brainstem, nerves, and blood vessels under a microscope. **k** Postoperative tumor bed, brainstem, nerves, and blood vessels under a microscope. 1, tumor; 2, accessory nerve; 3, vertebral artery; 4, brainstem; 5, C2 nerve; 6, cerebellum; 7, tumor bed

**Fig. 7:**
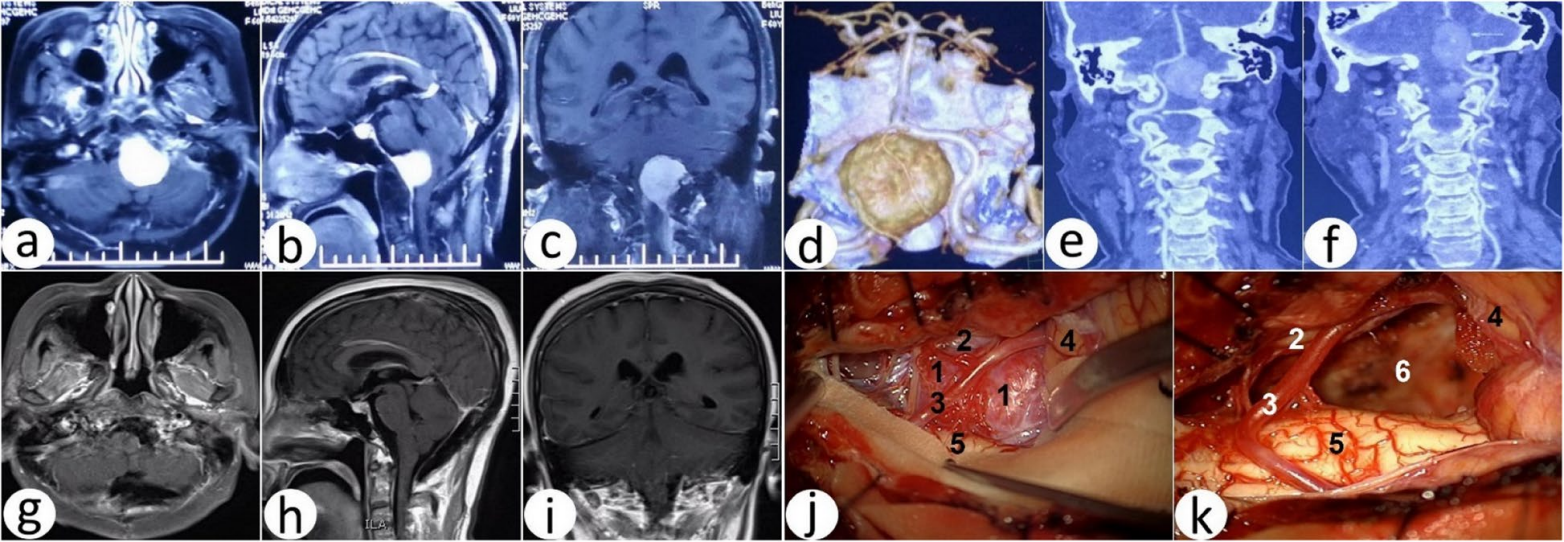
Comparison of preoperative and postoperative images of the FMM, which is located ahead of the brainstem and in the mix of vertebral artery. **a**–**c** Preoperative axial, sagittal, and coronal T1-weighted contrast enhanced. **d** Preoperative 3DCTA and tumor image reconstruction. **e**–**f** Preoperative right and left vertebral artery 3DCTA image reconstruction. **g**–**i** Postoperative axial, sagittal and coronal T1-weighted contrast enhanced. **j** Preoperative tumor, brainstem, nerves, and blood vessels under a microscope. **k** Postoperative tumor bed, brainstem, nerves, and blood vessels under a microscope. 1, tumor; 2, vertebral artery; 3, PICA; 4, cerebellum; 5, brainstem; 6, tumor bed

In this study of 76 cases, according to the relationship between FMMs and the brainstem and VA, the tumors were located ahead of the brainstem in 47 cases (A), and 10 cases were located superior to the VA (S) in which five cases underwent paracondylar FLA (P-FLA), three cases underwent basic FLA (B-FLA) (Fig. 6), two cases underwent supracondylar FLA (S-FLA), 29 cases were located inferior to the VA (I) in which 21 cases underwent B-FLA, 8 cases underwent transcondylar FLA (T-FLA), eight cases were mixed to VA (M) in which four cases underwent B-FLA (Fig. 7), two underwent T-FLA, and two underwent S-FLA.

**Fig. 8:**
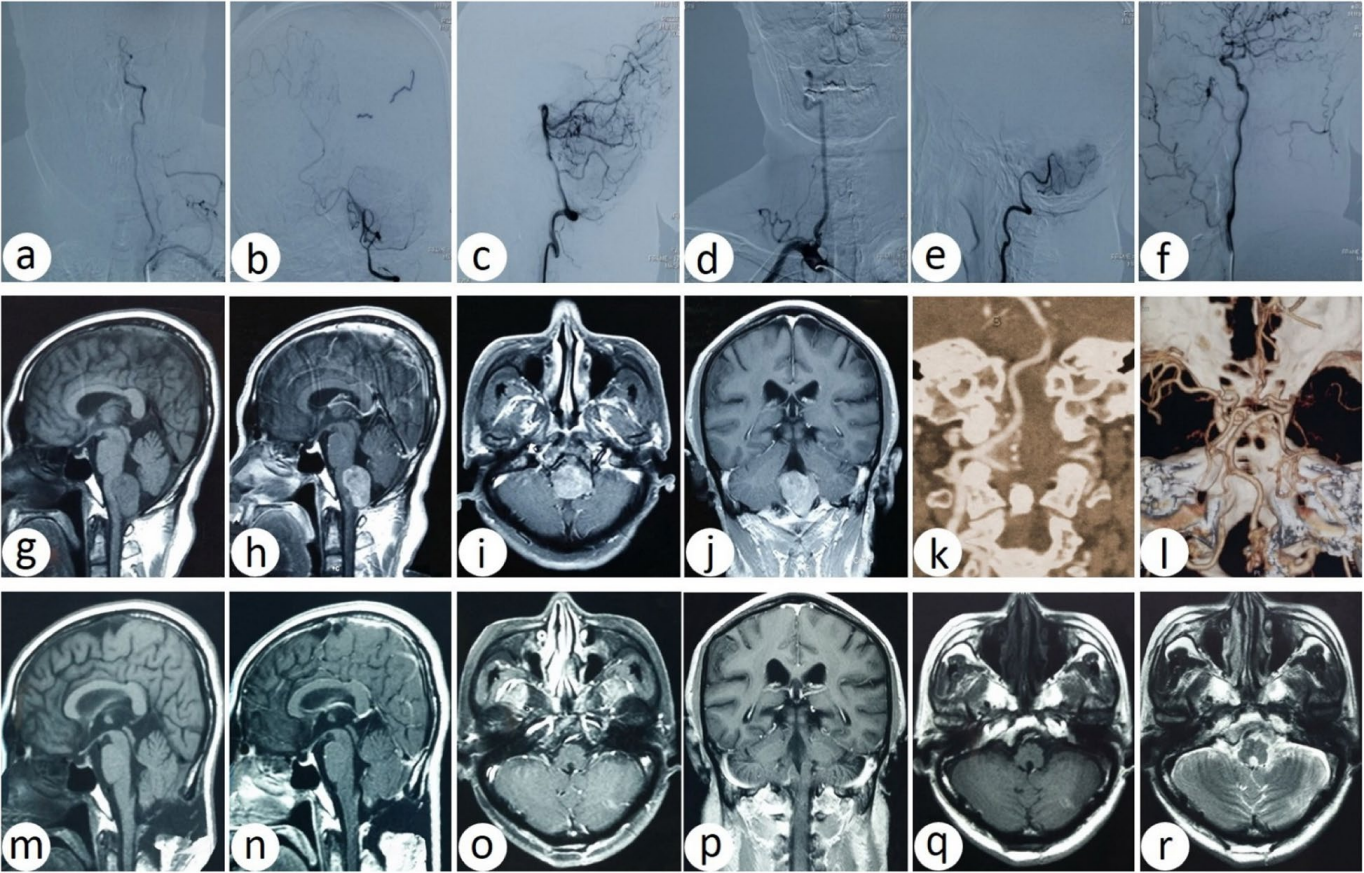
Comparison of preoperative and postoperative images of the foramen magnum meningioma, which is located in the back of the brainstem and superior to the vertebral artery. **a**–**c** Orthotopic, oblique, and lateral DSA of the left vertebral artery. **d**–**e** Orthotopic and lateral DSA of the right vertebral artery. **f** Orthotopic DSA of the right cephalic artery. **g**–**h** Preoperative sagittal T_1_-weighted MRI and contrast-enhanced MRI. **i**–**j** Preoperative axial and coronal T1-weighted contrast-enhanced MRI. **k**–**l** Preoperative left vertebral artery 3DCTA image and bilateral vertebral artery image reconstruction. **m**–**n** Postoperative sagittal T_1_-weighted and contrast-enhanced MRI. **o**–**p** Postoperative axial and coronal T1-weighted contrast-enhanced MRI. **q**–**r** Postoperative axial flair and T2-weighted MRI

Tumors were located at the back of the brainstem in eight cases (B), among which four cases were located superior to the VA (S) (Fig. 8), one case inferior to the VA (I), three cases mixed to VA (M), and all underwent a suboccipital midline approach.

**Fig. 9:**
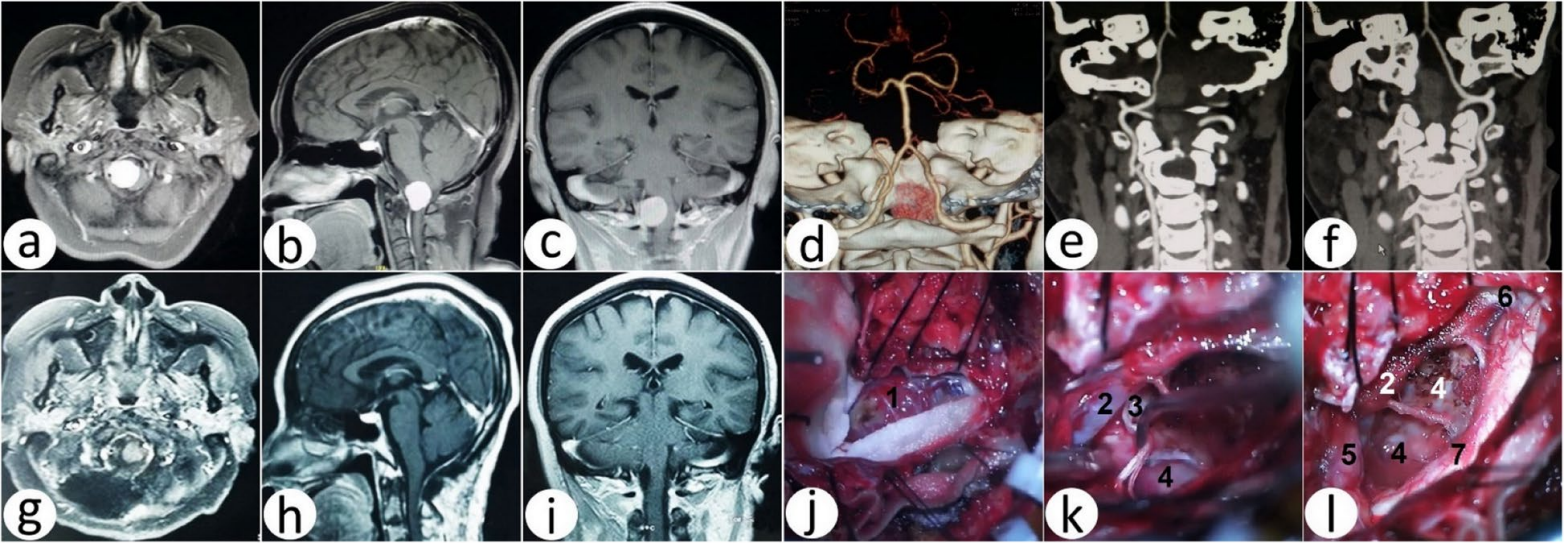
Comparison of preoperative and postoperative images of the foramen magnum meningioma, which is located on the side of the brainstem and in the inferior to the vertebral artery. **a**–**c** Preoperative axial, sagittal, and coronal T1-weighted contrast enhanced. **d** Preoperative 3DCTA and tumor image reconstruction. **e**–**f** Preoperative right and left vertebral artery 3DCTA image reconstruction. **g**–**i** Postoperative axial, sagittal, and coronal T1-weighted contrast enhanced. **j** Preoperative tumor under microscope. k. Postoperative tumor bed, vertebral artery, and hypoglossal nerve under a microscope. **l** Postoperative tumor bed, vertebral artery, brainstem, and C2 nerve under a microscope. 1, tumor; 2, vertebral artery; 3, hypoglossal nerve; 4, tumor bed; 5, cerebellum; 6, C2 nerve; 7, brainstem

Tumors were located on the side of the brainstem in 21 cases (S), among which five cases were located superior to the VA (S) (two cases underwent P-FLA, two cases underwent B-FLA, and one case underwent S-FLA); nine cases were located inferior to the VA (I) (two cases underwent B-FLA (Fig. 9), seven cases underwent a suboccipital midline approach), seven cases were located mixed to the VA (M), (five cases underwent B-FLA, and two underwent the suboccipital midline approach) (Table 2).

Among the 76 cases, there were 71 cases resected with Simpson grade 2 (93.42%). In three cases, including one case of meningioma recurrence, there were obvious brainstem adhesions in these three cases, and there was a thin layer of residual tumor tissue on the surface of the brainstem to protect it and resected with Simpson grade 3 (3.95%). Two cases (both were recurrent meningiomas, there were serious brainstem and VA adhesions in both cases, and it was very difficult to separate them) were resected with Simpson grade 4 (2.63%).

### Four triangles in operation

**Fig. 10:**
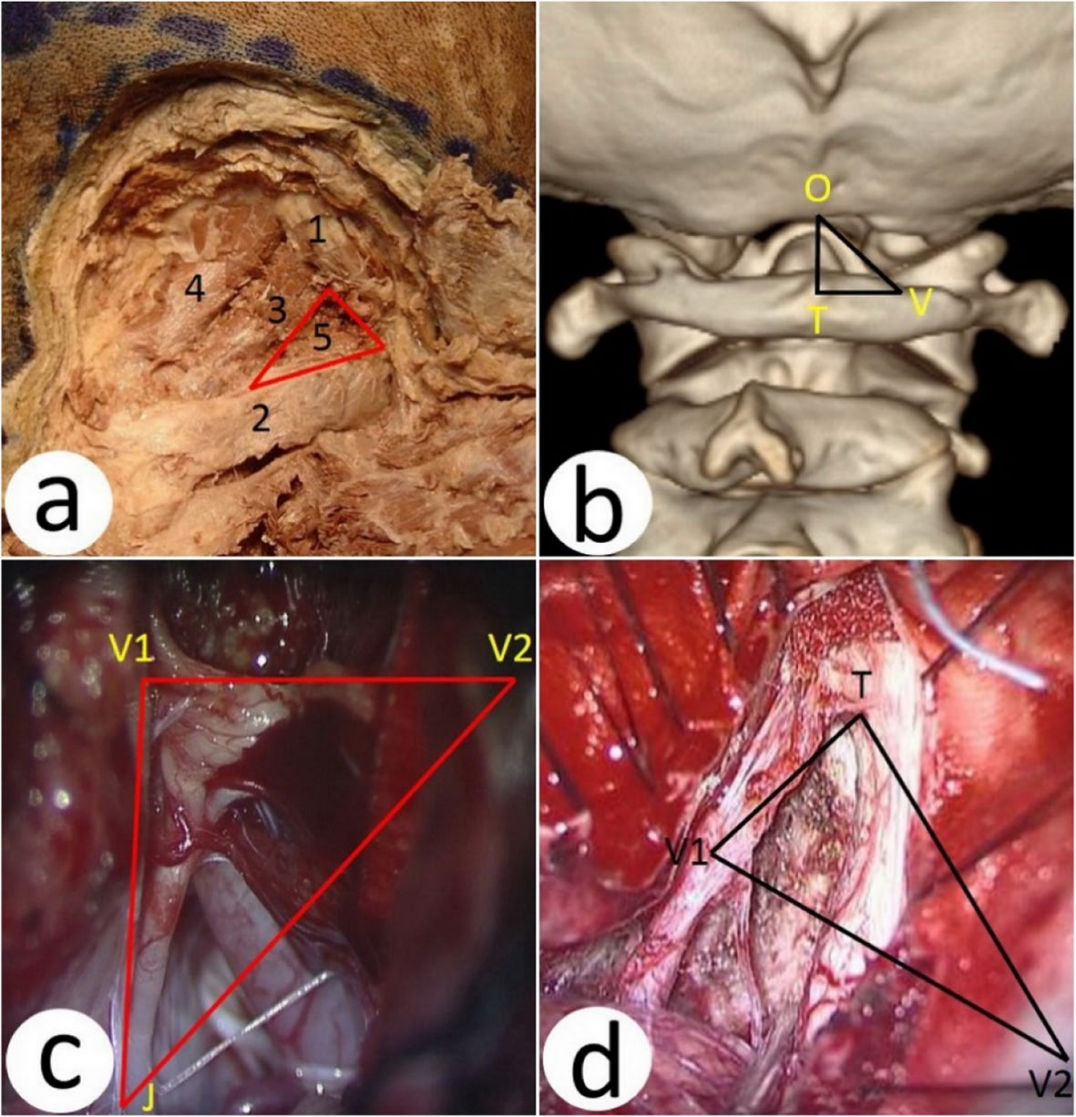
Four important triangles in operation (right side). **a** Triangle SOT: 1, oblique capitis superior; 2, oblique capitis inferior; 3, rectus capitis posterior major; 4, rectus capitis posterior minor; 5, vertebral artery. **b** Triangle VOT, V, point V3 segment of the vertebral artery entering the trouble side dura; O, opisthion; T, tubercula posterius. **c** Triangle JVV (triangle above the rectus capitis posterior minor): J, jugular foramen of the trouble side; V1, point V3 segment of the vertebral artery entering the trouble side dura; V2, the V3 segment of the vertebral artery enters the uninjured side of the dura. **d** Triangle TVV (triangle under the vertebral artery), T. inferior pole of a tumor; V1, point V3 segment of the vertebral artery entering the trouble side dura; V2, the V3 segment of the vertebral artery enters the uninjured side of the dura

We summarized four anatomical “triangles” of FMM and the main parts that need to be paid attention to in each subsurgical area to resect the tumor and protect the important structures in operation:

1. Triangle SOT: The suboccipital triangle, located in the suboccipital region, is a triangle surrounded by the suboccipital muscles. The triangle SOT is composed of the posterior musculi rectus capitis located in the inner upper bound, the superior musculi obliquus capitis located in the outer upper bound, and the inferior musculi obliquus capitis located in the outer lower bound. The base of the triangle is the posterior occipital membrane of the atlas and the posterior arch of the atlas. The V3 segment of the VA passes through the triangle, penetrates the posterior occipital membrane into the spinal canal, and then enters the skull through the foramen magnum of the occipital bone, continuing as segment V4 of the VA. The suboccipital nerve is the posterior branch of the 1st cervical nerve, which also passes through the triangle and penetrates between the VA and the posterior arch of the atlas and supplies the suboccipital muscle (Fig. 10a)
2. Triangle VOT: The triangle is made up of lines of three points, which are the point of the V3 segment of the VA entering the dura, the midpoint of the posterior margin of the foramen magnum (opisthion), and the tubercula posterius (Fig. 10b).
3. Triangle JVV (triangle above the VA): The triangle comprises three lines: the jugular foramen, the point of the left V3 segment of the VA entering the dura, and the point of the right V3 segment of the VA entering the dura (Fig. 10c).
4. Triangle TVV (triangle under the VA): The triangle comprises three lines: The tumor bottom, the point of the left V4 segment of the VA entering the dura, and the point of the right V3 segment of the VA entering the dura (Fig. 10d).

### Postoperative complications

Postoperative complications mainly included seven cases of hoarseness, hoarse drinking water choking, and dysphagia, five cases of hypodermic fluidification, four cases of pneumonia, three cases of poor cough reflex, three cases of neck and occipital numbness, three cases of incision infection, two cases of great occipital neuralgia, two cases of dyspnea, two cases of cerebrospinal fluid leakage, two cases of hydrocephalus, and one case of decreased muscle strength of the contralateral limb. The cerebral stem and spinal cord vasospasm was considered, which was relieved after 2 weeks of antivasospasm treatment. There were no surgical deaths (Table 1). No tumor recurrence or craniocervical instability was observed during the follow-up period of 3–106 months.

## Discussion

### Imaging evaluation and selection of surgical approach

#### Imaging evaluation

Preoperative MRI and enhancement imaging for FMM are necessary, and it is important to distinguish the tumor location, texture, and the relationship between the tumor and the dura. We named the classification ABS according to the relationship between a tumor and the brainstem on MRI imaging, and we think it is the most difficult to operate if the tumor is located ahead of the brainstem (A), followed by the side of the brainstem (S), and the back of the brainstem (B) in an actual surgical operation. It is helpful to predict the texture of a tumor and the space between a tumor and the arachnoid membrane, and it is instructive to design the surgical approach and evaluate the difficulty of tumor resection. For example, the long T2 image indicates a soft tumor; it is a relatively soft tumor that is easier to remove, and bone removal during the operation should not be too much if a tumor shows a long transversal relaxation time-weighted image (T2W), while the short T2 image is the opposite. Bone invasion, subluxation, and total dislocation of the atlantoaxial joint can be observed using 3DCT of the foramen magnum. In our experience, if the distance between the axial odontoid and the atlantoaxial odontoid is greater than 3 mm on the midsagittal plane, it is considered that the dislocation of the atlantoaxial joint exists, and the fixation of the dislocation should be performed after surgery. We named the classification SIM according to the relationship between FMM and VA based on combined head and neck 3D-CTA imaging, and we believe it is the most difficult to operate if the tumor is located in the mixed type (M), followed by superior to the VA (S) and inferior to the VA (I) in an actual surgical operation.

In addition, for the location at the back of the brainstem of the FMM (B), regular 3D-CTA imaging showed strong contrast enhanced, and there was a close relationship between FMM and blood vessels; a DSA examination needs to be performed to further distinguish the solid hemangioblastoma of the dorsal medulla oblongata [5, 11, 14, 20–22]. This is of great significance because there are essential differences in surgical preparation, operation scheme, operation difficulty, and prognosis of patients between these two diseases.

### The selection of surgical approach

There are a variety of extensions of FLA, including B-FLA, T-FLA, S-FLA, P-FLA, and extended FLA (E-FLA) [16, 23–28], and we think that the approach should be combined to analyze ABS and SIM classifications (Table 2).

### The main points during FLA

#### Flap formed

The skin flap of the FLA used in the study was adopted in an inverted L-shaped incision, and then, the skin flap was flipped with the cervical occipital muscles. Because the VA in the suboccipital triangle is protected by its surrounding venous plexus and muscles, the probability of injury is rare. If not necessary, the VA may not be exposed and turned over to reduce vascular injury and spasms caused by the extension of the VA [29, 30].

#### Bone removed

It is essential to remove the occipital condyle in FLA. The removal of the internal portion of the occipital condyle only increased the exposure by 7%, but the operating space increased significantly by 22% [19, 25, 31, 32]. The more ventral the lesion, the larger the range of the occipital condyle removal, but the range of removal of the occipital condyle was not beyond the hypoglossal canal; that is, it could not exceed the posterior 1/3 of the long axis of the occipital condyle. Some scholars believe that the ventral field of the BS has been improved by gradually pushing it backward in some long-term extramedullary subdural lesions, providing additional operating space. It is feasible to remove the ventral subdural tumor of the foramen magnum without removing the occipital condyle, and the disability rate could be reduced [15, 33]. Other studies believe that a small amount of the occipital condyle can be removed if the tumor is located in the ventral lateral of the brainstem and the superior cervical spinal cord because extra space would be exposed during the tumor resection process, and the tumor has created access pathways through the relationship with peripheral nerve and vessels [5, 34, 35]. During the operation, the ventral side of the brainstem and the high cervical spinal cord can be observed and exposed in a coronal plane of 45° or more, to reveal the relationship between the tumor and the medulla oblongata and cervical spinal cord, without important structures retraction, such as the medulla oblongata and cervical spinal cord, to effectively expose and remove the tumor [12, 36].

Among the 76 patients in this group, 54 patients were treated with FLA, including 31 cases of B-FLA, 12 cases of P-FLA, 9 cases of T-FLA (including five cases with the posterior 1/3 of the occipital condyle removed, four cases of less than 1/3), two cases of S-FLA, and no atlanto-occipital fusion.

#### Removal of the tumor

The operation was performed among the brainstem, posterior cranial nerves, C1, C2, and C3, and blood vessels under neural electrophysiological monitoring [11, 20, 22, 37–40]. Most tumors should usually be removed first within the tumor, the tumor root is cut off after the tumor volume is reduced, and the tumor tissues can then be removed piecemeal because the root of the tumor is adjacent to the running position of the VA and the tension of the tumor is high. Special attention should be paid to distinguishing and protecting the VA and its branches because their color is very similar to the color of the tumor, and they overlap with each other.

#### The contents and significance of the four triangles in operation

1. Triangle SOT: In this triangle, the content is the V3 segment of the VA. In this study, the FLA flap was flipped together with the occipital neck muscles, at the end of which was the position of the suboccipital triangle. When the posterior arch of the atlas is separated laterally, the VA in this area should be noted (Fig. 10a).
2. Triangle VOT: In this triangle, the contents are also the V3 segment of the VA, in which the average distance between the bottom line of the TV was 18.62 mm [41]. Therefore, it is important to understand the anatomy and relevant data of this location to guide the scope of the removal of the posterior atlas nodules during the operation and to protect the V3 segment of the VA [42] (Fig. 10b).
3. Triangle JVV: In this triangle, the contents are brainstem, tumor, V4 segment of the VA, posterior inferior cerebellar artery, glossopharyngeal nerve, vagus nerve, accessory nerve, hypoglossal nerve, etc. The triangle is the most important functional region with the most complex structure of the nerves and vessels. Any problem with the abovementioned nerves and vessels will show the corresponding clinical symptoms and manifestations. The S and part of M of the SIM classification will involve the above important structures. We must be very familiar with the anatomical position and function of each structure during the operation and strive to achieve functional reservation, preferably anatomical reservation (Fig. 10c)
4. Triangle TVV: In this triangle, the contents are the brainstem, high cervical spinal cord, tumor, accessory nerve, C1 nerve, C2 nerve, C3 nerve, etc. There are also many important nerves and vessels clustered in the triangle TVV, except for the V4 segment of the VA, which is easily confused with tumor tissue in terms of similar color and location. The nerves and vessels in this area also need to be preserved functionally, even anatomically. Although the hypoglossal nerve is not included in this region, it can also be seen on the abdominal side of the tumor when the tumor is removed completely if the tumor tissue grows upward and the VA is lifted upward, and attention must be paid to separate and protect it carefully (Fig. 6k). In addition, we do not advocate cutting off the C1, C2, and C3 nerves proactively; otherwise, complications after surgery may make patients very uncomfortable (Fig. 10d).

### The main points during the suboccipital midline approach

The incision was performed strictly according to the median, and the foramen magnum and posterior arch of the atlas were opened gently. The latch of the medulla oblongata is the respiratory regulation center; therefore, pathological adhesion and double-click electrocoagulation should be carefully considered. Attention should be paid to the protection of the bilateral VA, posterior inferior cerebellar artery (PICA), and branches. The upper and lower vermis must be moderately dissected. The cerebrospinal fluid flow of the four ventricles should be ensured after tumor resection.

### Postoperative complications and management

In the group of 76 cases, the function was significantly improved after administration of a nasogastric diet within 3 months of postoperative hoarseness, hoarse drinking water choking, and dysphagia; hypodermic fluidification was cured after puncture and compression bandage; pneumonia was cured after effective anti-inflammatory therapy; poor cough reflex was recovered 1 month after tracheostomy and regular sputum aspiration; neck and occipital numbness was relieved after expectant treatment; incision infection was cured after incision dressing and anti-inflammatory treatment; great occipital neuralgia was relieved after oral carbamazepine treatment; dyspnea was recovered after tracheostomy and ventilator-assisted respiration for 2 weeks, and tracheal cannula was extracted 6 weeks later; cerebrospinal fluid leakage recovered after 2 weeks of lumbar cisternal drainage; intracranial infection was recovered after 2 weeks of lumbar cisternal drainage and 4 weeks of effective anti-inflammatory therapy; hydrocephalus were recovered after ventriculoperitoneal shunt; and the muscle strength of the contralateral limb decreased. The cerebral stem and spinal cord vasospasm were considered, which was relieved after 2 weeks of antivasospasm treatment and functional training.

## Conclusions

The location is deep, and the operation risk is high in the FMM. It is an objective indicator to distinguish the surgical difficulty of occipital foramen area lesions of ABS and SIM classifications, and it is necessary to gradually understand and master the characteristics and effectiveness of the subdivision, which plays an important role in preoperative evaluation and prognostic analysis. The four “triangles” we summarized in the surgical area contain all important nerves and blood vessels in the foramen magnum area, and it is of great significance to master the contents, their mutual position relationship with the tumor, and the variable anatomical structure for the success of the operation.

## Data Availability

Please contact author for data requests.

## Abbreviations

FMM: Foramen magnum meningioma
VA: Vertebral artery
FLA: Far lateral approach
KPS: Karnofsky performance score
CT: Computed tomography
MRI: Magnetic resonance imaging
3DCTA: Three-dimensional computer tomography angiography
DSA: Digital subtraction angiography
T1W: Longitudinal relaxation time-weighted images
T2W: Transversal relaxation time-weighted images
NICU: Neurosurgery intensive care unit
PICA: Posterior inferior cerebellar artery
